# Human Pathophysiological Adaptations to the Space Environment

**DOI:** 10.3389/fphys.2017.00547

**Published:** 2017-08-02

**Authors:** Gian C. Demontis, Marco M. Germani, Enrico G. Caiani, Ivana Barravecchia, Claudio Passino, Debora Angeloni

**Affiliations:** ^1^Department of Pharmacy, University of Pisa Pisa, Italy; ^2^MedLab, Institute of Life Sciences, Scuola Superiore Sant'Anna Pisa, Italy; ^3^Department of Electronics, Information and Biomedical Engineering, Politecnico di Milano Milan, Italy; ^4^Fondazione Toscana G. Monasterio Pisa, Italy

**Keywords:** microgravity, space radiation, endothelium, stress response, aging, pre-nosological medicine, bed rest, visual acuity

## Abstract

Space is an extreme environment for the human body, where during long-term missions microgravity and high radiation levels represent major threats to crew health. Intriguingly, space flight (SF) imposes on the body of highly selected, well-trained, and healthy individuals (astronauts and cosmonauts) pathophysiological adaptive changes akin to an accelerated aging process and to some diseases. Such effects, becoming manifest over a time span of weeks (i.e., cardiovascular deconditioning) to months (i.e., loss of bone density and muscle atrophy) of exposure to weightlessness, can be reduced through proper countermeasures during SF and in due time are mostly reversible after landing. Based on these considerations, it is increasingly accepted that SF might provide a mechanistic insight into certain pathophysiological processes, a concept of interest to pre-nosological medicine. In this article, we will review the main stress factors encountered in space and their impact on the human body and will also discuss the possible lessons learned with space exploration in reference to human health on Earth. In fact, this is a productive, cross-fertilized, endeavor in which studies performed on Earth yield countermeasures for protection of space crew health, and space research is translated into health measures for Earth-bound population.

## Introduction

Physiology under extreme conditions uncovers reactions of the human body that may advance our understanding of limits for a healthy organism and may also shed light on premonitory signs of disease. Spaceflight (SF) physiology is no exception. During the last 50 years of manned space exploration studies have shown that humans can adapt to space and can remain productive for up to 1 year and possibly longer.

Many of the basic problems of space medicine—hypoxia, dysbarism, thermal support, acceleration, and response to great altitudes—have been studied by aviation and diving medicine long before the first human SF. In fact, flight, diving, and space traveling all involve pressure changes, forced changes in body position, controlled breathing sources, and dependence on life support equipment (Barratt, 2008). However, space flight poses unique medical problems due to prolonged exposure to a combination of stressful stimuli, such as acceleration forces, radiation, and weightlessness. In particular, the latter condition is a critical feature of SF and has effects on human physiology which were quite unexpected at the beginning of space exploration.

To appreciate the uniqueness of SF conditions, one should consider that a static gravitational field of 9.81 m/s^2^ and a protective atmosphere are major factors that have made Earth amenable to life. The gravity force, always present with a fixed direction, has influenced the development of all organisms since the dawn of life (Anken and Rahmann, 2002). It was a major contributor to biological adaptations from water to land, by forcing ancestral organisms to develop complex systems for stability, fluid regulation, gravity sensing, and locomotion. In fact, every single cell may sense changes in gravity and convert mechanical stimuli into biochemical signals (Ingber, 1997). In Low Earth Orbit (LEO), astronauts are still exposed to about 90% of ground level gravity force. However, the spacecraft speed counterbalances the gravitational force and consequently astronauts are in a free-falling state resulting in an apparent weightlessness. In space, many physical and biological changes occur that highligth the importance of gravity in the evolutionary course, while coincidentally revealing unexpected links between microgravity, disease onset and aging. In fact, the adaptive response to microgravity by a healthy human has many features in common with aging, as both induce the decline of almost every body system.

In the 1970s, with three sequential Skylab missions of increasing duration (28, 56, and 84 days), each with three astronauts, enough data were collected to establish the foundation of space physiology (Dietlein, 1977; Buckey, 2006). From these preliminary results showing negative calcium balance with bone density loss, muscle atrophy, cardiovascular and hematic changes, metabolic, endocrine, and sleep disturbances, space physicians concluded that astronauts undergo rapid senescence in space. Their symptoms, in fact, were similar to those of the elderly (Vernikos and Schneider, 2010). However, this conclusion lost credit as soon as it was realized that astronauts could recover after returning to Earth. Systematic rehabilitation data are not always available and procedures are not standard, but the fact that anomalies subside in due time points to a significant difference from the normal aging process. Nevertheless, this coincidence of symptoms and the possibility that a longer stationing in space may have greater impact keeps the issue of accelerated aging at the fore front (Michel et al., 2007).

An additional issue is the possible impact of radiation exposure during SF. Outside the natural shield of the atmosphere and the Earth geomagnetic field, astronauts are exposed to heavy ions and proton fluxes, far higher than those experienced at most locations on Earth. Despite the limited number of crew members available for an analysis of space radiation effects, it is assumed that the perception of light flashes being reported by these individuals in a dark environment results from the action of heavy ions on the retina. Other long-term ocular damage may result from altered body fluid distribution in microgravity. The latter is of particular interest as it may provide insights on a possible combined impact of radiation exposure and microgravity.

## Weightlessness and fluid redistribution as stress factors in space

### Effects on the cardiovascular system

The cardiovascular system holds the capacity to adapt to vastly different environmental and metabolic conditions. Physiological adaptation to postural changes relies on multiple, coordinated mechanisms such as the Starling-type and those operated by the endocrine and autonomic nervous systems (ANS). Gravity is a crucial factor in fluid distribution and has played an enormous role in shaping the evolution of the cardiovascular system. In upright posture, gravity determines a pattern of fluid distribution with higher arterial pressure in the feet (200 mmHg) and lower pressure in the head (70 mmHg) relative to the heart (100 mmHg). In space, this gradient is lost. Blood redistribution toward the head causes altered responses of baroreceptor, nervous and endocrine systems (Katkov and Chestukhin, 1980). As a result, within few minutes of microgravity exposure, astronauts suffer from a syndrome, known as “space motion sickness,” which includes anorexia, vomiting, nausea, headache and malaise, gradually solved over 48–72 h. Dramatic physiological rearrangements due to head-ward body fluid shift occur under chronic microgravity exposure, from weeks to month, as well. However, the cause-effect relationship between fluid redistribution and space motion sickness remains not completely understood. An increase in cerebral spinal fluid and intracranial pressure has been theorized, but never demonstrated (Simanonok and Charles, 1994). Parasympathetic overstimulation, triggered by abnormal vestibular activity in microgravity, may play a crucial role, causing vomit, nausea, and baroreceptor desensitization. The interplay between vestibular and parasympathetic systems is currently under investigation (Clément et al., 1992).

Cardiovascular (mal)adaptation to microgravity is also responsible for acute orthostatic intolerance on returning to earth gravity, which represents an important problem encountered by humans once they are adapted to microgravity: low vascular resistance and hypoadrenergic responses, related to a dysfunction of central integration of baroreflex afferent input as a result of SF, are considered the putative pathophysiological mechanisms of this phenomenon (Meck et al., 2004).

The best experimental setting for exploring acute and chronic cardiovascular adaptation in microgravity is represented by space missions. Nonetheless, precious experimental data and countermeasures against microgravity-dependent physiological adaptations have been collected from on-earth experiments. These include, for example, prolonged bed rest protocols and head-down tilt experiments for simulation of long-term microgravity exposure and free-fall parabolic flights (Limper et al., 2014), for acute simulation of microgravity.

Considering the complexity of the integrated neural and endocrine regulatory mechanisms that control the cardiovascular system, we will analyze the impact of SF on their operation in the following paragraphs.

#### Reflex mechanisms

Blood redistribution early during microgravity produces engorgement of the central circulation, sensed by mechanoreceptors which activate autonomic offloading and volume regulating reflexes. As a result, there is a vasodilation and pooling of blood in the viscera and tissues and initial renal fluid and salt loss. Most adaptations occur within 6–10 h of SF.

Concerning heart rate (HR), values during SF have been reported as higher, lower, or unchanged from preflight values. Significantly, different authors have reported an evident fall in HR, in both short- and long-term missions (Fritsch-Yelle et al., 1996; Verheyden et al., 2009). Such reduction is consistent with a rise in arterial pressure being sensed by neck baroreceptors due to cephalic blood redistribution. A recent 14-day mission involving three Chinese astronauts failed to show changes in mean HR, but nevertheless HR excursions increased compared to pre-SF measurements (Liu et al., 2015).

Similarly, conflicting data exist on mean arterial pressure (MAP). Several authors have claimed that microgravity decreases MAP (Fritsch-Yelle et al., 1994; Baevsky et al., 2007; Verheyden et al., 2009), while others have reported the opposite, at least during short-term SFs (Hughson et al., 2012). This discrepancy is likely due to differences in the definition of the pre-flight baseline as well as in a variety of countermeasures and study protocols being adopted in-SF. Recently, thanks to a regular exercise program, six male astronauts being stationed for 2–6 months in the International Space Station (ISS) could redress any change in systolic and diastolic pressure, and presented normal MAP (Hughson et al., 2012).

The baroreflex acts in a coordinated way on both heart and blood vessels to control blood pressure (BP), with the effects on the heart occurring in parallel to those on systemic vascular resistance (SVR). Indeed, a decreased SVR may contribute to the increase in CO reported during SF. However, data from a long-term ISS mission showed a non-significant reduction in SVR (although this was possibly due to the high variability in the experimental group; Hughson et al., 2012). Shear blood stress in vessel remodeling might help interpreting the impact of SF on SVR, but the literature is rather elusive and contradictory on this issue. For years experimental observations were based on animal models exposed to simulated microgravity. The initial hypothesis was that vascular smooth muscle cells would suffer from deconditioning in space and undergo atrophy similar to that observed in skeletal muscles, thus resulting in loss of tone and hypotension (Trappe et al., 2009). However, several experiments performed on Earth on the hindlimb-unloaded rat model demonstrated that the outcome of vessel remodeling processes changes according to the anatomic region involved. At 1 g, vessel walls stressed by BP become hypertrophic, while low BP leads to wall atrophy. Furthermore, the lumen increases in the carotid and the basilar arteries, while becomes narrowed in the hindlimb rat vessels, like the femoral and the anterior tibial arteries (Zhang, 2001). Some studies have reported reduced vasoconstriction in hind limb and abdominal arteries (Ma et al., 1997; Zhang et al., 2001), while others have reported basilar artery and common carotid vasoconstriction (Purdy et al., 1998). In light of that, the attendant effect on SVR may be small and variable. Therefore, it is important to consider both vasoconstriction and vasodilation responses to microgravity when evaluating any change in SVR.

NO release may represent an additional factor impacting on SVR. In fact, 20-day tail suspension resulted in the up-regulation of inducible nitric oxide synthase (iNOS) in thoracic aorta and heart, and in the over-expression of neuronal nitric oxide synthase (nNOS) in the brain and kidney (Schrage et al., 2000).

Recently, it was shown that mice exhibit an impaired vasoconstrictive response of mesenteric arteries and veins to norepinephrine (NE), KCl, and caffeine during SF, due to a decreased response of the adrenergic receptors and a reduced influx of Ca^++^. However, these results did not match with any macroscopic structural change of the arteries (such as lumen and wall thickness), nor did they show impairment in passive mechanical properties (Taylor et al., 2013). Collectively, these data indicate that both structural changes and molecular adaptations to microgravity may suppress the vasoconstrictive response to ANS, thus reducing SVR. Consequently, the arterial pressure control may become impaired and this causes orthostatic hypotension upon return on Earth.

#### Endocrine mechanisms

Bearing in mind the key role of the kidney in BP regulation, in space the upward redistribution of blood volume with the concomitant decrease of a pressure gradient should reduce kidney perfusion. In turn, a lower than normal pressure within the renal artery should lead to a greater release of renin and, ultimately, to a more marked activation of the renin-angiotensin-aldosterone system (RAAS).

Still, kidney perfusion has been found normal in space, along with increased plasma filtration, diuresis, and natriuresis (Leach et al., 1975). One should note however that more recent studies show a marked reduction of diuresis and natriuresis in space with NE, RAAS, and antidiuretic hormone (ADH) increasing through unknown mechanisms (Grigoriev et al., 1994; Drummer et al., 2000; Christensen et al., 2001).

A drastic weight-loss of the astronauts proved to be unrelated to the increased diuresis and natriuresis (Christensen et al., 2005). Imbalanced fluid distribution and atrophy of the antigravitational muscles (e.g., postural muscles and lower limb muscles) may facilitate fluid and albumin accumulation within the muscle interstitial space. An increase of glomerular filtration and by extension of diuresis and natriuresis, is ascribed to reduced colloid osmotic pressure. Accordingly, long-term BP adjustment in space may be mainly sympathetic-dependent, since RAAS activation is not due to altered kidney perfusion but rather to higher NE plasma concentration. In space, the colloid osmotic pressure due to plasma proteins is the only factor that prevents fluid extravasation. Moreover, microgravity reduces interstitial fluid removal by the lymphatic system, since this drainage depends on tissue deformation and local hydrostatic pressure (Hargens et al., 2013). This situation leads to oedema formation, particularly evident as a facial congestion (Parazynski et al., 1991).

Recent work has investigated the actual role of barorereflex and endocrine mechanisms in long-term microgravity exposure. Data collected between May 2007 and December 2009, before, during and after a 6-month mission to the ISS, showed no change in the baroreflex response slope while adopting an accurate countermeasure program (Hughson et al., 2012). Indeed, these data (Hughson et al., 2012) differ from those collected in a 10-day mission (Drummer et al., 2000) and confirm some previous findings while disagreeing with other investigations (Eckberg et al., 2010; Verheyden et al., 2010). From this evidence, it seems possible that over an extended period other mechanisms, possibly hormone release, may progressively displace the baroreflex in the control of BP. However, the gradual adjustment of the human body to weightlessness may involve other mechanisms besides the baroreflex and endocrine systems that on Earth are the main players in the operation of the cardiovascular system.

In summary, despite some contradictory results from different groups employing diverse research protocols, a consensus may be reached over the fact that the circulation of blood becomes more efficient in microgravity. Specifically, a lower HR supports the body's energy and perfusion requirements and, notwithstanding the higher stroke volume, the heart works less since it does not operate against gravity. Although, microgravity *per-se* does not seem to be a stress factor, the rapid adaptive changes being triggered by fluid redistribution, while appropriate for space, cause a significant deconditioning and orthostatic hypotension upon return to Earth. These dynamic phenomena occurring during SF provide the opportunity to investigate pathophysiological mechanisms underlying deconditioning and orthostatic hypotension, regardless of its etiology.

### Effects on the endothelium: a molecular view

The endothelium is a spatially diffuse organ, composed of a single layer of cells (the endothelial cells, ECs) that covers the inner surface of blood and lymphatic vessels (Fishman, 1982; Augustin et al., 1994). ECs function ensures vascular integrity and homeostasis and regulates a variety of essential physiological processes. Conversely, endothelial dysfunction is involved in a variety of vascular pathologies (e.g., atherosclerosis, hypertension, thrombosis) as well as in cardiovascular anomalies frequently found in a sedentary and elderly population (Cines et al., 1998). EC senescence is characterized by alterations in adherens junctions and leads to increased vascular permeability. This phenomenon mostly involves small blood vessels. Vascular permeability is central to the progression of different diseases linked to aging (Oakley and Tharakan, 2014), with pro-inflammatory (e.g., Tumor Necrosis Factor alpha, TNF-α, and TNF-Related Apoptosis-Inducing Ligand, TRAIL) and patently inflammatory (e.g., histamine, thrombin, Vascular Endothelial Growth Factor, VEGF) stimuli causing cytoskeleton disorganization (Kumar et al., 2009; Sawant et al., 2011).

Significantly, head down tilt bed rest (HDBR) studies have provided evidence of an increase in circulating ECs (CECs) in case of diffuse tissue damage (Demiot et al., 2007). ECs are most susceptible to variations in gravitational loading (Augustin et al., 1994; Cines et al., 1998), as exemplified by studies in an animal model (Xianyun et al., 1997) showing an increase in CECs in response to simulated microgravity.

ECs isolated from vessels of different size and location in the body are morphologically and functionally heterogeneous (Monahan-Earley et al., 2013). Since ECs from the microvasculature cover a surface 50 times larger than that of all large vessels lumped together, the endothelium of small blood vessels has a marked impact on systemic homeostasis. Its importance is evident from the modulation of inflammation to wound healing and tissue repair. A microvascular endothelial dysfunction is likely to play a significant role in osteoporosis, muscle atrophy and in the cardiovascular deconditioning being reported by astronauts. The same sequence of events may also occur in healthy volunteers during long-lasting HDBR (Demiot et al., 2007; Coupé et al., 2009; Morrison et al., 2014).

Several studies have reported the response of ECs to either simulated or real microgravity, although they mostly involve models of macrovascular ECs, HUVEC or their derivative EA.hy926 (Maier et al., 2015). Of note, several devices may simulate microgravity on Earth (including 2D clinostat, random positioning machine, RPM, rotating wall vessel, RWV, and diamagnetic levitation; Herranz et al., 2013), but the resulting gravity vector is averaged to near zero over time and is not neutralized (Herranz et al., 2013). On the other hand, microgravity can be obtained through sounding rockets, drop towers, or parabolic aircraft flights, albeit over very short periods, while aboard the ISS or other spacecraft samples are exposed to real microgravity (Herranz et al., 2013) for a considerable amount of time.

In simulated microgravity, HUVEC showed increased migration and proliferation (Carlsson et al., 2003), along with down-regulation of interleukin-1α (IL-1α), an antagonist of endothelial proliferation, and up-regulation of heat shock protein 70 (Hsp70). Conversely, up-regulation of apoptotic signals (e.g., Bax, Fas-L) occurs in porcine aortic endothelial cells (PAEC) cultured in RPM.

NO is an important effector for many signaling pathways and a potent vasodilator responsible for vascular homeostasis. Increased NO synthesis following NOS up-regulation is a common trait of EC adaptation to microgravity (see also Reflex Mechanisms above). It might have a crucial role in the endothelial response to microgravity since its level increases in both microvascular ECs from murine (1G11; Cotrupi et al., 2005) and human (HMEC-1; Mariotti and Maier, 2008) source as well as in macrovascular HUVEC (Shi et al., 2012) and EAhy926 (Siamwala et al., 2010) cells of human origin.

Responses to simulated microgravity suggest substantial differences between microvascular and macrovascular ECs, but the interpretation of findings is made difficult by intrinsic variations in cell types and ground simulators among cell biology studies in microgravity. Indeed, the same experimental model may generate different responses when challenged in diverse devices or by space microgravity. Although not evident morphologically, these differences emerge at the molecular level (Grimm et al., 2002; Pietsch et al., 2013; Ma et al., 2014; Warnke et al., 2014).

In Summer 2015, we dispatched samples of human microvascular ECs HMEC-1 (a reductionist representation of human dermal microvascular endothelium) to the ISS (Balsamo et al., 2014). Still in progress, integrated analyses of their transcriptome, methylome, and morphology in real microgravity should shed light on the adaptive response to the space environment and, possibly, should also uncover differences with ECs of macrovascular origin.

A new type of EC, type H, mediates neo-angiogenesis, and osteogenesis in bone (Kusumbe et al., 2014). The decline of type H cells and the concomitant reduction of osteoprogenitor cells could offer a convincing explanation for the loss of bone mass during aging. If so, the activation of specific molecular pathways promoting type H vessel formation may pave the way to therapeutic osteogenesis. In this light, it would be of interest to characterize the response to microgravity by type H ECs and by extension to evaluate their possible role in the response of the human body to SF.

In sum, it is clear that gravity has shaped the physiological properties of different components of the cardiovascular system. However, the impact of SF on blood vessel functions and specific properties is far from being explained by the response of cells and tissues to just microgravity alone.

### The eye, microgravity, and vision

The eye is a small organ whose function relies on the coordinated operation of its optical, vascular, epithelial, and neural components. In space, the fluid redistribution caused by microgravity affects the blood supply to the eye with an impact that depends on specific facets of its vascularization. The evolution of initial fluid redistribution into long-lasting effects on eye anatomy and function depends on SF duration (Mader et al., 2011). Blood supply to the retina and the posterior pole of the eye is via the ciliary arteries and the central retinal artery branches of the ophthalmic artery, a branch of the internal carotid artery, although variations are frequent (Hayreh, 2006). The retina has an oxygen consumption rate per unit weight higher than the brain (Ye et al., 2010), consistent with the high metabolic rate of photoreceptors (Demontis et al., 1995, 1997). To support retinal oxidative metabolism (Wang et al., 2010), the choriocapillaris of the choroidal vascular bed have evolved as a system with large fenestrated capillaries (i.e., high flux and permeability) and low oxygen extraction (Linsenmeier and Padnick-Silver, 2000). Furthermore, the choroidal circulation may absorb the heat generated by the focusing of the infrared component of light on the posterior pole of the eye (Parver et al., 1980), a role of high relevance in the foveal region (Parver, 1991). Similarly, blood supply to the eye supports the secretion of the aqueous humor by the ciliary epithelium, thus generating an intraocular pressure (IOP) to set eye tonicity. This process likewise satisfies the metabolic needs of eye tissues lacking a vascular bed, such as the lens and the cornea. In turn, IOP affects the perfusion pressure (PP) of the choroidal circulation that depends on the difference between blood pressure in the ophthalmic artery and IOP. In humans, the occurrence of autoregulatory mechanisms (Riva et al., 1981, 1997a,b) prevents increases in IOP from translating into corresponding changes in choroidal blood flow, despite their influence on PP. PP regulations are also apparent in response to HDBR protocols, with a persistent increase in ophthalmic arterial pressure, IOP and PP in response to transient changes in the size of retinal arterioles and venules (Baer and Hill, 1990). Optical coherence tomography (OCT), paired with angiography using near infrared fluorescent probes poorly absorbed by pigment epithelium such as indocyanine green, has significantly enhanced the functional evaluation of the choroidal circulation, otherwise not accessible using visible light (recently reviewed; Ferrara et al., 2015). Similar to retinal vessels, HDBR tilting increased choroidal thickness and IOP in proportion to the tilt angle, as assessed by OCT and indocyanine green angiography, while retinal thickness was largely unaffected (Shinojima et al., 2012). These results indicate that an upward shift in blood distribution may quickly affect eye vasculature.

Blood redistribution toward the head induced by microgravity is expected to affect eye vasculature by reducing arterial blood supply and slowing venous flow from the eye. It is important to remind that venous blood return from the head, neck and upper trunk is typically assisted by gravity, as it occurs via veins that lack both valves and muscular contraction, as opposed to the lower half of the body, where this mechanism provides the propulsive force that opposes gravity. In microgravity, a reduced blood flow return from head and neck to the heart is expected to raise the venous pressure and increase filtration at the capillaries causing, in turn, an increase in both intracranial pressure and IOP, consistent with face swelling mentioned above (see Endocrine Mechanisms).

Several papers report the effects of microgravity on eye function. Short exposures to microgravity during parabolic flights (20 s-long) were found associated with a significant increase (58%) in IOP and a non-significant decrease (4%) of retinal arteries size, indicating that the effects of microgravity on IOP take place on a short time-scale (Mader et al., 1993). An important issue is whether these rapid changes in the eye vascular bed affect its function.

Exposure to microgravity on a time scale of a few days, such as during shuttle flights, was associated with reduced near sight in about 23% of astronauts (Mader et al., 2011). Furthermore, 48% of astronauts returning from ISS long-duration SFs reported near sight vision reduction (Mader et al., 2011). Detailed investigations in astronauts that reported visual problems upon returning from 6 month-long SFs indicate the occurrence of ocular damage. A hyperopic shift was found in 6/7 astronauts, with posterior eye pole flattening and choroidal folding in 5/7 (Mader et al., 2011).

MRI investigations of orbital and intracranial effects of microgravity were carried out in 27 astronauts at a variable time after their return from space, while pre-SF control data were not available (Kramer et al., 2012). However, in 8 of them, the analysis included measurements carried out between two consecutive SFs and in 7/27 (26%) globe flattening was present. This study also documented swollen and bent optic nerve sheath in 26/27 (96%) astronauts. Importantly, evidence of optic nerve protrusion in 4/27 (15%) and pituitary dome concavity in 3/27 (11%) astronauts support the hypothesis of an increase in intracranial pressure (Kramer et al., 2012).

Overall, these findings recall those found in patients affected by idiopathic intracranial hypertension (Jacobson, 1995). Specifically, the elevation of fluid pressure within the skull may lead to an increased volume of choroidal vessels, shifting the fovea along the anterior-posterior axis with ensuing shortsightedness, but this hypothesis awaits confirmation (Nelson et al., 2014). Swelling of choroidal vessels may also damage the papilla, the site of optic nerve emergence from the eye. However, despite the expected increase in intracranial pressure in all the flying astronauts (those exposed to microgravity, as opposed to those that trained but did not travel to space), globe flattening was found in at least one eye in only 7/27 astronauts. Moreover, changes in visual acuity were reported to occur after 3 weeks up to 3 months in microgravity (Mader et al., 2011), well beyond the initial increase in cranial pressure from blood redistribution (Tatebayashi et al., 2002), making problematic a straightforward link between the immediate increase in cranial pressure and late visual impairments. Furthermore, the persistence of these anatomical changes and visual problems several months after return to Earth, i.e., after removing the initial increase in intracranial pressure, suggests that the structural damage possibly results from additional events triggered by intracranial hypertension during long-term exposure to microgravity (Taibbi et al., 2013), rather than by hypertension *per-se*.

The finding that visual problems associated with prolonged exposure to space are present only in a fraction of astronauts suggests that genotype also plays a role in the frequency with which persistent functional and anatomical damage in response to microgravity may appear. Interestingly, those astronauts that developed eye and vision problems (Zwart et al., 2012) had reduced folate and increased homocysteine levels, despite similar nutrition before, during and after SFs. Genetic polymorphisms in methylene-tetrahydrofolate-reductase (MTHFR), the gene coding for a key step in folate synthesis, are frequent and associated with reduced enzymatic activity. Therefore, differences in folate levels based on genetic backgrounds may account for part of the variability in the propensity of astronauts to develop eye problems in microgravity. However, despite this clear association, we lack a mechanistic link between folate deficit and the probability of developing eye problems in microgravity.

### Effects on the musculoskeletal system

Prolonged exposure to microgravity affects the musculoskeletal system, with the loss of bone and muscle mass attributed to both reduced use and perfusion changes (Hargens et al., 2013). Skylab missions showed an increase in bone resorption markers (n-terminal telopeptide, NTX, and hydroxyproline) and a significant reduction in Ca^++^ balance. Bone mineral loss was higher in sites supporting the body weight in normal gravity, like lumbar spine, femoral neck, and trochanter, pelvis, calcaneus, and leg, whereas arm bones were not affected (LeBlanc et al., 2007).

Soyuz and Mir missions confirmed the Skylab results, adding novel insights into bone marker change in cosmonauts. Levels of osteocalcin, a structural protein of the bone, slightly but significantly increased in blood samples collected at day 14 in space, but significantly decreased after 110 days in space (LeBlanc et al., 2000). Osteocalcin drastically increased upon return to Earth. Data from long-lasting American and Russian missions (*n* = 60, between 4 and 6.5 months) demonstrated that 92% of crewmembers suffered 5% loss of bone mineral density (BMD) in at least one skeletal site (LeBlanc et al., 2000). For the sake of comparison, the rate of BMD loss occurring during a 6-month stay on ISS is similar on average to that occurring between the 5th and 6th decade of life on Earth.

Osteoclast activity increases and major bone resorption occurs in 14-day long, −6° HDBR studies, with control subjects showing increased level of resorption markers in blood and urine compared with subjects undergoing vibration training as countermeasure. The analyzed markers included urine levels of c-terminal telopeptide (CTX) and NTX, in addition to blood levels of bone alkaline phosphatase (bALP) and Procollagen type I N-terminal propeptide (PINP; Smith et al., 2005).

In reference to bone metabolism, the levels of vitamin D, bALP, osteocalcin, calcitonin, parathyroid hormone (PTH), and Ca^++^ have been investigated. Results from the Mir18 mission showed a significant decrease in 1,25(OH)_2_-vitamin D and its precursor 25(OH)-vitamin D. Calcitonin levels were not affected either in space or back on Earth. The observation that PTH values rapidly increase upon return to Earth (Smith et al., 1999) indicates detrimental mass bone restoration, as PTH promotes bone resorption and elevates blood Ca^++^ levels by activating osteoclasts. However, PTH acts on kidneys too, to increase Ca^++^ and decrease ${\text{PO}}_{4}^{3-}$ reabsorption (Lee et al., 2009), suggesting that bone loss in microgravity may be due mainly to impaired kidney function and vitamin D production, rather than to a significant calcitonin and PTH imbalance. Therefore, reduced vitamin D level may decrease Ca^++^ fixation in bones and reabsorption in kidneys, causing increased Ca^++^ excretion despite normal levels of calcitonin and PTH (Smith et al., 1999). Vitamin D level increases later on, upon a prolonged re-exposure to gravity, to promote net bone fixation.

Perfusion remodeling during SF may affect bone mass, and unbalance the ratio between osteoblastic and osteoclastic activity. Data on perfusion-dependent effects on muscle mass are only available from tail-suspended mice (a simulation of weightlessness). Tail suspension was performed for 10 min, 7 days, and 28 days. Femoral and tibial perfusion were reduced with 10 min of treatment, and blood flow to the femoral shaft and marrow were further diminished with 28 days of treatment. Correspondingly, the mass of femora and tibiae was lowered with 28 days of tail suspension. In this condition, the femur was hypoperfused both in acute and chronic simulated microgravity, while the tibia showed a perfusion increase at the distal bone region only, at least in acute treatment. However, the authors (Colleran et al., 2000) may not have considered that tibia fuses with fibula in mouse (Moss, 1977) and the vascular anastomosis of tibial and fibular arteries provides blood supply to the tibia. Interestingly, it was shown that blood flow to the skull, mandible, and humerus increases only acutely (i.e., upon acute perfusion). It is possible that in chronic conditions aortic pressure increases, due to the blood shift from the hindlimbs to the forelimbs and head, and adjusts within a longer period by vasoconstriction of the most perfused arteries (Colleran et al., 2000).

There is high variability in results regarding extension and grade of muscle atrophy, which may ensue from different durations of muscle unloading but also relate to pre-flight muscle size in a non-linear way (Belavý et al., 2009). For instance, when comparing the plantar flexors with the knee extensors, atrophy was more pronounced in the former, possibly because during locomotion on Earth a higher load stresses the plantar flexors than the knee extensors. Consequently, unloading in space causes more damage in plantar flexors than in the extensors. The damage does not derive from fiber loss, but rather from a reduction in their size and protein synthesis.

Exactly what type of muscle fiber suffers the most damage from exposure to microgravity is still unclear, due to controversial results from human and mouse models (Belavý et al., 2009). Even though type I fibers seem to be the most affected in both species, the underlying mechanism may be different. In humans exposed to 5-week HDBR, protein anabolism was severely affected, as shown by the decreased synthesis of myofibrillar proteins and collagen along with an unchanged catabolism, unlike the mouse model that showed considerable protein breakdown (de Boer et al., 2008).

To explore the mechanism underlying the loss of anti-gravitational muscle mass in response to prolonged disuse in humans, the fascicle length and pennation angle were analyzed in the gastrocnemius and in the vastus lateralis at the end of 5 weeks of horizontal BR. In both muscles, the reduction of the pennation angle was quite remarkable, and mirrored the loss of sarcomeres, both in parallel and in series. Interestingly, these parameters were not affected in biceps brachii, an upper limb muscle (de Boer et al., 2008). At the functional level, data on neuromuscular activity registered by electromyography (EMG) to investigate the plantar flexor activity showed a 35–40% reduction after 90–180 days in space (Lambertz et al., 2001). Of note, these data differ from those showing an increased EMG activity in the tibialis anterior and soleus, without changes in the gastrocnemius medialis (Edgerton et al., 2001).

High individual heterogeneity might partially account for such differences. The muscular parameters of cosmonauts were studied focusing on their maximal voluntary contraction (MVC), maximal shortening velocity index (VImax) and musculotendinous stiffness index (SIMT), collecting data pre- and post-SF. Despite a significant decrease in MVC and an increase in VImax and SIMT, the number of observations was small and there was high inter-individual variability (Edgerton et al., 2001).

In conclusion, it is not surprising that a varying degree of muscle loss results from different duration of microgravity exposure. These considerations may have a major impact on rehabilitation after injuries, especially in aging subjects, highlighting the need to focus on those muscle groups more prone to mass loss in response to unloading.

### Effects on the respiratory system

Microgravity-induced respiratory modifications have been studied for decades, focusing on several parameters that characterize respiration and ventilation (Baranov et al., 1992; Prisk, 2000; Prisk et al., 2006). Different studies are difficult to compare due to environmental bias (for example, during the Skylab missions in the 1970s, data were collected from astronauts exposed to the hypobaric and hyperoxic environment; Sawin et al., 1976) and to the variability of experimental protocols (which included pre-, in- and post-SF measurements; Baranov et al., 1992). Furthermore, indirect experimental procedures were used to estimated gas exchange and the ventilation-perfusion ratio.

Studies from Spacelab missions 1 and 2 on lung ventilation and perfusion showed that microgravity causes a slight increase in respiratory frequency and a reduced physiological dead space thanks to homogenous blood redistribution in lung vessels. Data from the same missions described a reduction in tidal volume (Donnelly et al., 2010), which was confirmed by other studies (Prisk et al., 2006). Data collected from a 6-month stay on ISS (where the environment is normoxic), failed to show significant modifications in gas exchange in space. However, there was a significant reduction in O_2_ consumption and CO_2_ production at 2 and 4 months (Prisk et al., 2006). The reason why this happens remains unknown (Prisk, 2014). A possible explanation needs to consider multiple concomitant factors. First, the reduction of muscle work due to reduced gravitational loading. Second, the difference in perfusion and ventilation between upper and lower lung regions, which decreases and may require less frequent and deep ventilation by the respiratory muscles. Altogether, these effects are expected to reduce the metabolic rate.

Changes in thorax wall and respiratory mechanics are among the first aspects to address when considering respiration in space. In microgravity, the contribution of the abdomen to the tidal volume increases, while rib cage expansion is reduced (Wantier et al., 1998). Studies in the 1990s suggested that microgravity decreased vital capacity (VC; Venturoli et al., 1998), forced vitality capacity (FVC), and peak inspiratory and expiratory flows (Baranov et al., 1992). The authors proposed that microgravity could weaken respiratory muscles, compromising the respiratory function. However, more recent data questioned these concepts: apart from a significant early increase during the mission's first 2 months, VC and FVC at 4 and 6 months were not statistically different from pre-SF values (Prisk et al., 2006). Peak inspiratory and expiratory flows showed the same trends. Prisk et al. showed a significant reduction in maximum inspiratory pressure (MIP) at functional reserve capacity (FRC) and residual volume (RV) during the entire mission (Prisk, 2000). Maximum expiratory pressure (MEP) at total lung volume was significantly reduced at 2 and 4 months but showed a trend toward full recovery at 6 months. MEP at FRC was not affected. Notably, the same authors reported a significant reduction in tidal volume between in-SF respiration and steady respiration on-Earth (Prisk, 2000). As already mentioned, this may be due to different lung perfusion in space: since blood redistributes upward, lung vessels should receive more blood, resulting in more efficient gas exchange and therefore lead to a lower excursion between inspiration and expiration at rest.

Remarkably, FRC in space reduces significantly to around 500 ml (Elliott et al., 1994). This reduction is explained directly by microgravity: since FRC is a direct measure of the balance between lung collapse and the outward expansion of the thoracic chamber, in space the contribution of the abdominal content weight to thorax expansion becomes negligible, therefore FRC falls (Prisk, 2014). RV measured after forced expiration decreases significantly in space. In fact, on Earth alveoli at the bottom of the lungs deflate more easily than the alveoli at the top, causing the retention of a larger amount of air in the upper lung regions. In space, this does no longer occur and therefore RV is lower than on Earth (Elliott et al., 1994).

These results suggest that the respiratory system undergoes significant adaptation after prolonged exposure to blood redistribution in microgravity. These changes develop over several weeks, possibly indicating that adaptation occurs through anatomical rather than functional changes, a finding relevant to the impact of prolonged bed rest on elderly subjects or chronically ill patients.

## Radiation effects

In addition to weightlessness, a main environmental factor acting upon the human body in space is represented by cosmic radiation. Far from Earth, beyond the Van Allen radiation belt, the astronauts of long-term missions to Moon or Mars would be exposed to remarkable doses of radiations of two kinds: solar particle events (SPE) and galactic cosmic radiations (GCR), which cause acute and late morbidity respectively. SPE occur in space with different intensity according to periodic oscillations of solar activity and contribute to total irradiation for a maximum 5% (Cucinotta et al., 2013). GCR consist mainly of protons and high atomic number and energy nuclei (HZE, 1%), establishing a qualitative difference between cosmic and Earth radiation spectra, which consists of α-, β-, and γ-rays.

A 3% risk of exposure-induced death (REID) at the upper 95% confidence interval is considered the maximum acceptable risk for a space mission (Cucinotta and Durante, 2006). Future long-duration missions, including landing on Mars, need to take this limit into account because the estimated impact of radiations for a mission to the red planet lasting 460–780 days exceeds the REID limit (Cucinotta et al., 2013). However, these data lack accuracy since they are based on predictions for cardiovascular and ischemic heart disease computed from a meta-analysis of studies concerning atomic-bomb survivors and radio-exposed workers (Kennedy, 2014). In addition, while small animals and human cell cultures have been exposed to simulated GCR, the effect of HZE particles was not investigated, although this aspect is quite relevant indeed. In fact, HZE particles are qualitatively different from the protons of GCR and exert a stronger effect on biomolecules, resulting in complex DNA damage and higher amounts of reactive oxygen species (ROS) formation, which in turn leads to chronic oxidative stress and increases drastically genomic instability (Kennedy, 2014).

Radiation effects on the human body can be classified either as acute or long-term. The first may cause the astronauts to suffer from acute radiation sickness, including vomiting, nausea, and a falling blood count (Donnelly et al., 2010); the latter corresponds to cancer development. The risk of suffering from acute radiation poisoning is quite low during internal vehicle activity, also during long-term missions. Nonetheless, consequences of acute irradiation could decrease the white blood cell count, and break epithelial layers thus resulting, for example, in translocation of bacteria and their products in the intervillous district of the ileum. T-lymphocyte decrease, vomiting, and nausea, skin damage with loss of blood vessels density under the dermis could occur as well (Cucinotta et al., 2013; Kennedy, 2014). Disseminated intravascular coagulation is among the most serious acute effects of chronic exposure to cosmic radiations.

Concerning long-term effects, experiments conducted on mice proved that 0.5–3 Gy protons or 0.5 Gy ^56^Fe exposures reduced overall survival and caused malignant lymphoma and Harderian gland tumors. In experimentally irradiated mice, pre-malignant and malignant lesions of myeloid origins were observed, with proton and γ-ray-induced accumulation of p53, along with increased ROS generation and deregulation of the extra-cellular matrix compartment (Barcellos-Hoff et al., 2015).

When exposed to radiation in microgravity, an organism may in principle suffer from a diminished capability of DNA repair systems, which physically consist of enzymes transported through the cell to exert their role in the nucleus whenever necessary. In fact, there is at the moment evidence of the combined effects of both microgravity and radiations on different organs and organ systems, such as the eye, the cardiovascular, and the immune systems. Although, we will not discuss here the latter (well-reviewed in Crucian et al., 2014), still we want to remark that several other factors such as isolation, confinement, and disrupted circadian rhythms in general add to microgravity and radiation to generate the complex phenotype of a space-exposed organism.

### Radiation effects on the eye

During SF, cosmic radiations are expected to affect all organs to some extent. The eye is particularly susceptible to cosmic radiations, as it lacks the protections warranted to inner organs by the skin, with its layer of dead keratinocytes, or to the brain by the skull. Not surprisingly, the first evidence of the effects of cosmic radiation on the human body came from Apollo 11 lunar module pilot Buzz Aldrin (Pinsky et al., 1974), who reported the occurrence of light flashes (LF) upon dark-adaptation. Most astronauts have later reported the occurrence of LF when dark-adapted. There is evidence that multiple components of cosmic radiation may cause LF (Casolino et al., 2003). Depending on the energy of impinging particles, LF may result from retina stimulation by photons generated via Cerenkov's effect by particles traversing the vitreous (Fazio et al., 1970) or the lens (McAulay, 1971). However, an additional mechanism may explain HZE-triggered LF. Hydroxyl radicals generated by HZE interaction with water (Yamaguchi et al., 2005) may cause the peroxidation of polyunsaturated fatty acids (PUFA) ensheathing rhodopsin molecules in the disk membrane of rod outer segments (reviewed in Catalá, 2006). Evidence for PUFA peroxidation induced by the exposure to high-energy carbon ions via reactive hydroxyl radicals has been gathered both *in vivo* and *in vitro*. Furthermore, the annihilation of two peroxy radicals leads to the emission of a photon, which may isomerize a rhodopsin molecule (Narici et al., 2012, 2013). Due to the high amplification of the phototransduction cascade in retinal rods, isomerization of a rhodopsin protein by a single photon is sufficient to lead an individual to LF perception (Baylor et al., 1979). According to this model, rod photoreceptors work as a sensitive probe to detect light emitted in response to a chain reaction triggered by cosmic radiation via the generation of highly reactive radical species, thus signaling the occurrence of nearby damaging molecules. However, the generation of reactive radical species by HZE may also take place in other retinal neurons, although it may go unnoticed due to the lack of light-sensitive probes for photons emitted during radical annihilation. In fact, the electrophysiological response of mice exposed to both light and ^12^C ions suggest that charged particles do not simply mimic the light response at the rod level, but rather may interfere with the processing of light signals downstream of rods (Carozzo et al., 2015). Consistent with this notion, increased apoptosis along with ganglion and amacrine cell damage are indicators of inner retinal injury in space-flown mice. This damage correlated with increased accumulation of lipid peroxidation byproducts and up-regulated expression of genes involved in response to oxidative stress as well as in mitochondria-associated apoptotic pathways (Mao et al., 2013). The lack of damage at the site of origin of LF (outer retina) may result from the protection afforded to rods by rhythmic disk shedding, which removes the outer segment tip damaged by HZE-generated free radicals (recently reviewed in McMahon et al., 2014).

The notion that HZE may trigger lipid peroxidation and ultimately lead to the activation of the phototransduction cascade in rods and LF perception by astronauts is supported by multiple and independent evidence gathered both *in vitro* and *in vivo*. However, some discrepancies remain. In particular, it is unclear how to reconcile the role of HZE-triggered lipid peroxidation with the observation that LF frequency is about twice as much when traveling toward the Moon than on the way back. Although the sensitivity of astronauts to LF may decrease during the first few days in orbit, the underlying mechanisms and their link with lipid peroxidation are unclear. A second issue is the large inter-individual variability in sensitivity to LF (Pinsky et al., 1974). In fact, while some astronauts report the occurrence of LF as soon as they became dark-adapted (i.e., when the rod system takes over vision) and refer to LF as an annoying phenomenon that may interfere with their ability to rest (Fuglesang et al., 2006), others are far less sensitive. At the extreme, the Apollo 16 command module pilot Thomas K. Mattingly did not report LF at all, possibly because of his poor night vision (possibly confirming the role of rods in LF perception). However, the occurrence of intermediate sensitivities to LF in astronauts with normal rod-mediated vision may indicate a role of additional, as yet unknown, factors. By analogy with the eye damage caused by microgravity (see The Eye, Microgravity, and Vision), genetic polymorphisms may play a role in setting the sensitivity of astronauts to LF perception in response to HZE exposure.

## Lessons learned for/from space research

### Countermeasures for space from bed rest studies

Ground-based studies represent an essential opportunity to investigate human physiology in simulated microgravity, and thus to test the effectiveness of potential countermeasures for preventing or mitigating the undesired physiological changes associated with SF, mentioned in the above paragraphs.

At the beginning of the human SF era in 1961, head-out water immersion was used to simulate weightlessness on the ground, limited to short periods (6–12 h). In the early 1970s, head-out dry immersion (i.e., immersion with an impermeable elastic cloth barrier between subject and thermo-neutral water, thus providing an absence of mechanical support of specific zones during immersion—supportlessness), was proposed (Shulzhenko and Vil-Vilyams, 1975) and then widely used, in particular by Russian scientists (Navasiolava et al., 2011). Gradually, the model of HDBR became the method of choice for studying the effects of prolonged musculoskeletal unloading on the ground. Based on the sensation of astronauts after long-duration SF, HDBR was considered more appropriate for simulating physiological changes of SF than horizontal BR, and −6° HDBR was found to be the best compromise among possible bed inclinations (Atkov and Bednenko, 1992). Placing healthy volunteers in bed became the model of choice for inducing and studying the effects of prolonged SF and for testing potential countermeasures. While earlier studies were more focused on the role of inactivity resulting from HDBR and related exercise countermeasures to restore normal physiological function (Sandler and Vernikos, 1986), subsequent research aimed at exploring new approaches for developing comprehensive, more efficient countermeasures (Pavy-Le Traon et al., 2007). However, the lack of standardized measures from different HDBR campaigns, conducted in different locations and by different space agencies, precluded drawing an overall conclusion on the efficiency of the various tested countermeasures. To overcome these limitations, in the context of the Roadmap for European Countermeasure Research with Bed Rest, in 2006 the European Space Agency (ESA) took the lead in defining a standardized framework for future HDBR studies. Fixed durations were defined for short-, medium-, and long-term HDBR, as well as the duration of ambulatory and post-HDBR period. In addition, a crossover design with one control and several treatment groups, with a washout period in between, was defined for short- and medium-term HDBR. To facilitate direct comparison among studies, a set of physiological measures (Bed Rest Core Data) and related times of examination was defined, thus providing standards for cardiovascular, nutrition, exercise, bone, and muscle, neuro-vestibular and psychology assessment, on top of other experimental variables proposed by different research groups. The application of this standardization started with the BR-AG1 study in 2010 (Table 1). In this study, +1 Gz artificial gravity (AG) at heart level elicited by short-arm centrifuge, was applied as daily countermeasure using two different protocols (30 min continuously, and 6 × 5 min intermittent) in 12 male volunteers enrolled in a cross-over design. The two protocols did not show differences in aerobic power (peak VO2) after HDBR compared with the control condition, while the 6 × 5 min protocol was more effective in preserving orthostatic tolerance after HDBR. However, neither protocol attenuated plasma volume loss (Linnarsson et al., 2015), nor prevented changes in left ventricular function (Caiani et al., 2014). Compared to the 30 min protocol, the intermittent AG protocol resulted in increased MVC capability in the knee extensor and plantar flexor muscles (Rittweger et al., 2015), lower adrenocortical stress responses (Choukèr et al., 2013), and fewer neurovestibular symptoms (Clément et al., 2015).

**Table 1:** Updated list of ESA-sponsored bed rest campaigns since 2000.

**Name**	**Year**	**Place, subjects**	**PRE (d)**	**HDT (d)**	**POST (d)**	**Intervention**	**HDT angle**
LTBR 01-02	2001/2002	Toulouse, 25 males	15	90	15	1) Flywheel exercise 2) Bisphosphonate	−6°
STBR 01-02	2001/2002	Cologne, 9 males. Cross-over	9	14	3	Caloric variations in nutrition, Amino acid infusion	−6°
BBR	2003/2004	Berlin, 20 males	3	56	6	Vibration exercise	0°
WISE	2005	Toulouse, 24 females	20	60	20	1) Combined resistive exercise, aerobic exercise, Lower Body Negative Pressure 2) Nutritional supplement	−6°
BBR2-2	2007/2008	Berlin, 24 males	9	60	7	1) High-load resistive exercise (RE) 2) RE+ whole-body vibration	−6°
BR-AG1	2010	Toulouse, 12 males Cross-over	5	5	5	1) Artificial gravity by daily short-arm centrifuge for: 30 min 2) Intermittent 6x 5 min	−6°
SAG	2010/2011	Cologne, 10 males Cross-over	5	5	5	1) Locomotion replacement training 2) Upright standing	−6°
MEP	2011/2012	Cologne, 10 males Cross-over	7	21	6	Combined supplementation of 0.6 g whey protein (WP)/kg body weight (BW) and 90 mmol potassium bicarbonate (KHCO3)	−6°
MNX	2012/2013	Toulouse, 12 males Cross-over	7		6	1) Resistive Vibration Exercise (RVE) 2) RVE + Nutritional Supplement	−6°
RSL	2015/2016	Cologne, 24 males	14	60	14	Reactive jump	−6°
TBD	2017/2018	Toulouse, 20 males	14	60	14	Cocktail	−6°

*TBD, to be defined; d, duration (days) of the different bed rest phases (PRE, before; HDT, head-down tilt; POST, after HDBR). Cross-over, subjects repeated the study, first in the control group, then in the countermeasure one*.

In the SAG 5-d HDBR, daily 25 min upright standing as a countermeasure, compared to 25 min locomotion-replacement training including a combination of heel raising, squatting, and hopping exercise, were studied (Mulder et al., 2014). However, cardiovascular, bone and metabolic deconditioning persisted despite the applied countermeasures (Feuerecker et al., 2013).

The following MEP 21-d HDBR study focused on the beneficial effects of a nutritional countermeasure, constituted by supplementing high protein intake (1.2 g/kg body weight/d plus 0.6 g/kg body weight/d whey protein) with alkaline salts (90 mMol potassium bicarbonate/day). The results were unclear. The countermeasure resulted in marginal changes in structural myofibril properties, with a possible delay in hybrid fiber transition in some but not all subjects, together with no major shifts in the major proteolysis markers in biopsy material. Altogether, data suggested little if any proteolysis to occur above constitutive protein turn-over in disused human skeletal muscles (Blottner et al., 2014). The same nutritional countermeasure was also studied in the MNX 21-d HDBR in conjunction with a resistive vibration exercise (RVE).

In the 60-d RSL study, the efficacy of a countermeasure system that allows reactive jumps independently of gravitational forces (i.e., used in the horizontal position) was tested to prevent muscle and bone loss during HDBR. Starting in January 2017, another 60-d HDBR will study the effects of a novel nutritional countermeasure, consisting of a cocktail of several substances (530 mg/day of polyphenols + 168 mg of vitamin E with 80 μg selenium + 2.1 g omega-3). The cocktail is supposed to have an effect on blood antioxidant capacity, lipid metabolism, and muscle cell metabolism.

### Physical exercise programs to protect the musculoskeletal system

Physical exercise programs are the main countermeasure used pre-, in-, and post-SF to protect the musculoskeletal system. In the last decades, new solutions to mitigate the effects of microgravity on musculoskeletal atrophy were adopted. Remarkable progress from a rowing ergometer, used in the Skylab missions, to a much more complex motorized treadmill, used in the ISS, derived from a growing attention to this issue. Thanks to countermeasures, HR and the maximum O_2_ consumption seem not to change in short- and long-term missions, with suboptimal levels reached for O_2_ consumption.

Recovery on Earth appears to depend on the duration of a space mission. Moreover, post-SF responses and orthostatic tolerance change when exercises in an upright seated position are performed (Moore et al., 2010). However, despite these promising results regarding cardiac performance, other studies highlight that the current strategy is not sufficient to prevent severe loss of the anti-gravitational muscles like calf (Trappe et al., 2009). A program consisting of treadmill running/cycling and 3–6 days per week of moderate-intensity, resistive exercises was proposed (Caiozzo et al., 1996). Nonetheless, calf muscle showed serious damage: muscle volume, maximum voluntary contraction (MVC) and peak force declined after a 6-month stationing in space. Remarkably, the soleus was more affected than the gastrocnemius and the astronaut with the largest gastrocnemius experienced the more severe deterioration. Overall, the observed transition from slow MHC-I to fast MHC-IIa fibers was significant, despite individual variability. This phenomenon occurs both in animal (Caiozzo et al., 1996) and human models (Widrick et al., 1999). The reason for this transition is not clear, but might be linked to the upward redistribution of blood and the subsequent hypoperfusion and reduced oxygenation of the calf, a situation possibly causing reduced oxidative phosphorylation and eventually transition to oxidative/glycolytic metabolism typical of the fast MHC-IIa fibers.

The devices used by the crew were a bicycle ergometer, a treadmill, bungee cords, and an elastomer providing a resistance exercise, the Interim Resistive Exercise Device (iRED; Lamoreaux and Landeck, 2006). iRED is unsuitable for long-term weightless exercise programs (Lamoreaux and Landeck, 2006). Therefore, a new device called Advanced Resistive Exercise Device (ARED) was developed (Lamoreaux and Landeck, 2006). Experiments on the efficiency of ARED conducted on Earth gave positive results: there were not statistical differences between the ARED-trained and the free-weight exercise-trained groups regarding muscle strength, muscle volume, vertical jump height, and lumbar spine BMD gained in 16 weeks of exercises (Loehr et al., 2011). The machinery, now onboard the ISS, is expected to improve astronaut cardiovascular and musculoskeletal recovery.

Treadmill technology has recently made substantial progress too, aiming to overcome the limited weight load of the Subject Load Device (SLD) used in the old-model machinery through a novel Zero-Gravity Locomotion Simulator (ZLS). Simulations proved that this system can provide a ground resistance force (GRF) statistically comparable to the GRF provided by over-ground run (Genc et al., 2006).

Another suggestive solution to mitigate the adverse effects of microgravity is to simulate gravity itself. The best way to achieve this purpose is to exploit the centripetal force produced by a centrifuge. Applications are currently under investigations with interesting results. Recently, a study proved that 1 h per day of exposure to 2.5 Gz AG counteracts the adverse effects of a 21-day BR program on the muscle fibers of the vastus lateralis and gastrocnemius (Caiozzo et al., 2009). The torque-velocity of the knee extensor was better preserved after exposure to AG, compared to the HDBR control group, and the plantar flexor torque-velocity increased after the AG program, whereas parameters of the control group worsened. The muscle fiber cross-sectional area was preserved in the soleus, in opposition to the HDBR group. Investigations on the slow-to-fast fiber transition revealed the MHC-I fibers to be better preserved in the soleus of the AG group, but MHC-IIx fibers still increased in both the vastus lateralis and the gastrocnemius. However, centrifugation alone cannot revert the effects of microgravity in terms of oxygen uptake, HR, and pulmonary ventilation, despite cycle ergometer exercise (Greenleaf et al., 1999). An optimal solution could be represented by a combination of centrifugation with (i) intensive aerobic exercise for cardiovascular system protection and (ii) moderate exercise to prevent musculoskeletal system deterioration. However, the use of a small-radius centrifuge requires the rotation rate must increase to obtain a centripetal force comparable to the g-force. That may not be affordable by an astronaut exercising on the centrifuge. In addition, both the Coriolis Effect and the centrifugal force cause motion sickness, so further studies are required to determine the most appropriate exercise program integrated with AG exposure (Hargens et al., 2013).

Another tool to counteract the upward fluid shift in space might be represented by the Low Body Negative Pressure (LBNP) chamber. Exposure of the legs to a negative pressure causes interstitial fluid pressure fall in the legs and blood shift from the higher to the lower regions of the body. Results showed that −30 mmHg pressure increased transcapillary fluid transport by decreasing the interstitial fluid pressure and the plasma volume, without affecting the foot venous pressure. Moreover, after LBNP exposure, even though transcapillary fluid transport decreased again, the plasma volume remained lower. Although the plasma volume extracted from the blood flowing in the legs was not exactly quantified, the increased circumference of the leg during and after LBNP exposure suggested that the headward blood shift occurring in space had been effectively counteracted (Aratow et al., 1993). Thus, a crew might use the LBNP chamber during a resting period and alternate low-pressure exposure with aerobic exercise performed on a properly set centrifuge so as to maximize cardiovascular and musculoskeletal performance.

Space activity suits (SAS) or mechanical counter-pressure suits might represent another possible solution for the future. Those are spacesuits made to apply a steady pressure against the skin by means of skintight elastic garments. Several were designed and tested in the past. Recently, with the Soyuz TMA-18M 44S mission to the ISS, a new type of SkinSuit was tested in space (SkinSuit, 2015). It was developed over some years to provide simulated +Gz that increases gradually from the shoulders to the feet. SkinSuit aims to counteract the stretching of the spine in space, which causes the lower back pain experienced by 50% of astronauts early in their missions. The ability to control spinal elongation in space might also help reduce the risk of post-SF injury to intervertebral discs (IVD), which astronauts are at greater danger of experiencing upon return to Earth. In terrestrial life, the SkinSuit technology has the potential to help the elderly and many people with lower-back problems, in addition to improving support garments currently used for conditions like cerebral palsy.

Further, studies on whole-body vibrations investigated how RVE can ameliorate musculoskeletal performance in volunteers undergoing prolonged HDBR. In a cohort of 20 male subjects immobilized for 8 weeks and monitored during a 6-month follow-up (Belavý et al., 2012), a significant amelioration of the activity ratio of the lumbopelvic muscles and a not significant improvement of the co-contraction of the lumbopelvic extensors and flexors were documented. Another interesting research revealed tail-suspended mice to be affected by IVD hypotrophy and glycosaminoglycan/collagen ratio imbalance. When exposed to a vertical platform oscillating with a 90 Hz frequency, disc deterioration was mitigated. In particular, the disc height, the glycosaminoglycan content in the whole disc and typical dimensions of the nucleus pulposus were restored (Kennedy, 1998). It must be noted that in humans exposed to microgravity a biochemical rearrangement of the IVDs leads to disc hypertrophy, just opposite of what happens in mice (Holguin et al., 2011). However, the finding that RVE of the vertebral column can ameliorate the anatomic and biochemical status of the IVDs is still valid and might be addressed to humans too.

The space crew diet is supplemented with calcium, vitamins D and K, and bisphosphonates, a common postmenopausal drug also successfully tested in HDBR study to reduce bone reabsorption to minimize bone demineralization (Smith et al., 1999). Potassium citrate and PTH were also evaluated (Buckey, 2006; LeBlanc et al., 2007; Stein, 2013).

Interestingly, technologies and some medicines developed for SF have found application on Earth. Examples include a drug to improve physical condition and cognition with low side effects (phenotropil; Zvejniece et al., 2011), or a powerful antiseptic (miramistin; Vasil'eva et al., 1993). The most recent one is a medicine developed against astronaut bone loss and now used for the treatment of osteoporosis caused by menopause and chemotherapy (antibody against sclerostin protein; Rodent Research, 2016).

### Protection against irradiation

Two approaches can be useful to mitigate the adverse effects of space radiations. One is pharmacological and based on the prescription of antioxidants and other protective substances. Another one is mechanical, based on a protective physical barrier provided by novel materials—yet to be developed—to reinforce the spaceship (that we will not examine here).

Antioxidants can neutralize ROS generated by GRC and SPE particles. However, different molecules exert different antioxidative reactions, which render the administration of a combination of various antioxidants more protective. Thus, combined doses of L-Selenomethionine (SeM), sodium ascorbate, N-acetylcysteine, α-lipoic acid, vitamin E succinate, and coenzyme Q10 were administered both to cell cultures and mice (Kennedy, 2014). The most interesting results came from the *in vivo* model: after exposure to ^56^Fe ion (0.5 Gy), proton or γ-rays (both 3 Gy), the plasma total antioxidant status (TAS) decreased, but the administration of the previously cited antioxidant combination improved TAS. Moreover, use of the Bowman-Birk Inhibitor (BBI, a soybean-derived protease inhibitor) completely restored TAS. BBI mode of action is not clear yet, but it is used in chemotherapy to prevent cancer progression. BBI was also suggested to neutralize anti-inflammatory proteases, trypsin, and chymotrypsin (Kennedy, 1998). In another experiment, BBI, BBIC (Bowman-Birk Inhibitor Concentrate), SeM alone or in combination with ascorbic acid, coenzyme Q10, and vitamin E succinate protected HTori cells from transformation after HZE and proton radiation exposure (Kennedy et al., 2004). Hematopoietic tissue is most sensitive to irradiation, so the antioxidant activity was investigated in mice undergoing 1 and 8 Gy X-ray, γ-ray, and proton irradiation. Remarkably, antioxidants prolonged mouse life and improved the total count and neutrophil count in peripheral blood and partially prevented the degeneration of bone marrow cells even though the treatment could not rescue peripheral lymphopenia (Wambi et al., 2008). In addition to antioxidants, growth factors can be administered to stimulate granulocyte production and neutralize the lymphopenia reported after antioxidant treatment only.

Two forms of granulocyte colony stimulating factor (GCSF), filgrastim and pegfilgrastim, were given to mice exposed to SPE-like proton or γ-rays. Pegfilgrastim was found more effective for neutrophil protection (Romero-Weaver et al., 2013). On this basis, Pegfilgrastim, also known as Neulasta, was also administered to irradiated ferrets and pigs. Interestingly, the neutrophil count never decreased below the baseline level (i.e., before irradiation), suggesting that Neulasta can increase the number of circulating neutrophils in different animal models (Kennedy, 2014).

Chinese medicine inspired a suggestive solution for protecting lymphocytes from radiation-induced death. In China, the formula SWT (Si-Wu-Tang) was used in the past for treating patients suffering from radiation sickness after radiation accidents. The formula components were investigated and were found to be paeoniflorin, ferulic acid, tetramethylpyrazine, and fructose. The analysis of the physiological response induced by each of these compounds revealed that the only protective active principle for granulocytes and lymphocytes was, surprisingly, fructose. Irradiated mice treated with fructose showed higher levels of granulocytes and lymphocytes if compared to the irradiated untreated samples, and fructose supplementation of the diet before radiation exposure improved the outcomes (Romero-Weaver et al., 2014). Given that, these stimulating data need to be confirmed by further studies to better define the real impact on the immune system.

## Conclusion

Humans adapt to the hostile environment of space, characterized by the absence of gravity and chronic radiation exposure, through cardiovascular adaptation and drastic changes in metabolism, respiration, body mass, bone density, and muscle integrity.

From the analysis of published data, it appears that a reductionist approach (assuming one or another main challenging factor) cannot easily explain the impact of long-term exposure to SF conditions. Indeed, this review shows that the independent actions of either body fluid redistribution or radiation exposure may not account for the organism response to long-term SF. In addition, the role of hormonal stress response to weightlessness may contribute to shaping the adaptation of the human body to space although this aspect has remained largely unexplored. Indeed, increased cortisol levels during SF are expected to affect kidney operation, bone resorption, immunity, muscle loss, glycaemic control, endothelial response and may contribute to the discrepancies observed between short-and long-term SF and to the inter-individual variability as well.

An important side of human body adaptation to SF conditions is its relevance to human health in general. Analysis of SF impact on health may benefit aging people on Earth. Aging is a progressive, time-dependent physiological deterioration of the homeostatic response and adaptation to the external environment that makes the individual fragile and more vulnerable to diseases. Several molecular-oriented studies shed light on the molecular pathways involved in aging (Weinert and Timiras, 2003). On Earth, aging affects the cardiovascular, musculoskeletal, nervous, gastrointestinal, urinary and neuroendocrine systems (Boss and Seegmiller, 1981), with immune system impairment and an enhanced inflammatory activity (Montecino-Rodriguez et al., 2013). Remarkably, these changes occur in space 10-fold faster than on Earth (Vernikos and Schneider, 2010), allowing scientists to speculate that the astronauts may be an appropriate model for exploring the mechanisms of aging. SF may allow researchers overcoming limitations of studying precocious aging, for example in progeroid syndromes, and physiological aging in healthy primates that would require 15–30 years to be properly characterized (Le Bourg, 1999).

Ground simulations, HDBR studies among them (Sandler and Vernikos, 1986; Pavy-Le Traon et al., 2007; Vernikos and Schneider, 2010), have been essential to investigate and analyze observations made on space crews, about the influence of contributing factors, mapping time course of physiological changes and exploring biological mechanisms under controlled conditions. In turn, insights gained from HDBR studies found application in the health care of chronically-ill people.

Obviously, there are significant differences among the effects of SF, HDBR, and physiological aging. A possible conceptual context to compare, differentiate and understand them is offered by considering them as disorders of mechanotransduction. Ingber's tensegrity theory and research (Ingber, 1997) provide the framework for understanding how external and internal mechanical forces influence the living organism at molecular and cellular levels. His work reveals that “molecules, cells, tissues, organs, and our entire bodies use tensegrity architecture to stabilize their shape mechanically, and seamlessly to integrate structure and function at all size scales.” This theory could explain at a mechanistic level how intermittent mechanical forces applied externally, such as vibration (Rubin et al., 2002), movement, or exercise (Bachl et al., 1993), centrifugation (Vernikos et al., 2004), push/pull or intermittent tension (Pietramaggiori et al., 2007), can influence cell and tissue growth and function.

In this perspective, physiology of space and extreme environments might deepen our understanding of aging and/or medical disorders.

## Author contributions

DA conceived and designed the article and coordinated the manuscript preparation. GD contributed the chapter devoted to “Eye, microgravity, and vision” and rewrote extensively several sections; EC wrote the chapter “Countermeasures for space from bed rest studies.” CP contributed to the chapter “Weightlessness and fluid redistribution as stress factors in space.” All other chapters were contributed by MG, IB, and DA. All authors read, discussed, edited, and approved the whole work.

### Conflict of interest statement

The authors declare that the research was conducted in the absence of any commercial or financial relationships that could be construed as a potential conflict of interest.

## Abbreviations

ARED: Advanced Resistive Exercise Device
ADH: antidiuretic hormone
ANS: autonomic nervous systems
BP: blood pressure
bALP: bone alkaline phosphatase
BMD: bone mineral density
BBI: Bowman-Birk Inhibitor
BBIC: Bowman-Birk Inhibitor Concentrate
CTX: c-terminal telopeptide
CO: cardiac output
CEC: circulating ECs
EMG: electromyography
ECs: endothelial cells
eNOS: endothelial NOSl
FVC: forced vitality capacity
FRC: functional reserve capacity
GCR: galactic cosmic radiations
GCSF: granulocyte colony stimulating factor
HDBR: head-down tilt BR
HR: heart rate
HZE: high atomic number and energy nuclei
HMEC-1: human microvascular EC
iNOS: inducible nitric oxide synthase
iRED: Interim Resistive Exercise Device
IL: interleukin
ISS: International Space Station
IVD: intervertebral disc
IOP: intraocular pressure
SeM: L-Selenomethionine
LVETi: left ventricular ejection time index
LF: light flashes
LBNP: low body negative pressure
LEO: low Earth orbit
VImax: maximal shortening velocity index
MEP: maximum expiratory pressure
MIP: maximum respiratory pressure
MVC: maximum voluntary contraction
MAP: mean arterial pressure
SIMT: musculotendinous stiffness index
NTX: n-terminal telopeptide
nNOS: neuronal nitric oxide synthase
NE: norepinephrine
OCT: optical coherence tomography
PTH: parathyroid hormone
PP: perfusion pressure
PUFA: polyunsaturated fatty acids
PAEC: porcine aortic endothelial cells
PINP: procollagen type I N-terminal propeptide
RPM: random positioning machine
ROS: reactive oxygen species
RAAS: renin-angiotensin-aldosterone system
RV: residual volume
RVE: Resistive Vibration Exercise
REID: risk of exposure-induced death
RWV: rotating wall vessel
SWT: Si-Wu-Tang
SPE: solar particle events
SAS: space activity suits
SF: space flight
SVR: systemic vascular resistance
TAS: total antioxidant status
VEGF: Vascular Endothelial Growth Factor
VC: vital capacity.
